# Supplementary material for: Dietary Long-Chain Omega-3 Fatty Acids Are Related to Impulse Control and Anterior Cingulate Function in Adolescents
Source: Front Neurosci. 2019 Jan 9;12:1012. doi: 10.3389/fnins.2018.01012 (PMC6333752; doi:10.3389/fnins.2018.01012)
Supplement: Supplementary file 1 [file Data_Sheet_1.docx]

**Dietary long-chain omega-3 fatty acids are related to impulse control and anterior cingulate function in adolescents**

**(Supplement)**

**Valerie L. Darcey PhD MS RD, Goldie A. McQuaid, PhD, Diana H. Fishbein, PhD,**

**John W. VanMeter, PhD**

Main Effect of Task (Table 1 and Figure 1).

| **Region** | ***x*** | ***y*** | ***z*** | **Max *t*** | **Volume (mm3)** |
| --- | --- | --- | --- | --- | --- |
| Inferior frontal gyrus, left [BA 47] | -38 | 16 | -16 | -3.87 | 42 |
| Inferior frontal gyrus, right [BA 46] | 42 | 34 | 12 | 4.25 | 37 |
| Inferior frontal gyrus, right [BA 9] | 58 | 6 | 34 | 4.39 | 200* |
| Middle frontal gyrus, right | 34 | 30 | 30 | 3.84 | 36 |
| Cingulate gyrus, left [BA 24] | -8 | -8 | 40 | 4.28 | 50 |
| Middle frontal gyrus, left | -50 | 22 | 38 | 3.97 | 24 |
| Cingulate gyrus, right [BA 24] | 14 | -16 | 42 | 4.14 | 21 |
| Precentral gyrus, right | 38 | -10 | 44 | 4.20 | 38 |
| Supplementary Motor Area, left  [BA 6/4/24] | -4 | -20 | 58 | 5.21 | 246* |
| Middle frontal gyrus, right | 36 | -2 | 60 | 4.16 | 80 |
| Precentral gyrus, left | -18 | -26 | 60 | 3.57 | 10 |

Table 1. MNI Coordinates of local maxima for activation during successful inhibitions (Correct No-Go>Incorrect No-Go) (main effects) (cluster defining threshold ke=10, uncorrected p=0.001, df 86). *Group-level statistical maps were small volume corrected (SVC) for multiple comparisons within the PFC. Cluster surviving FWE correction at p<0.05 denoted.


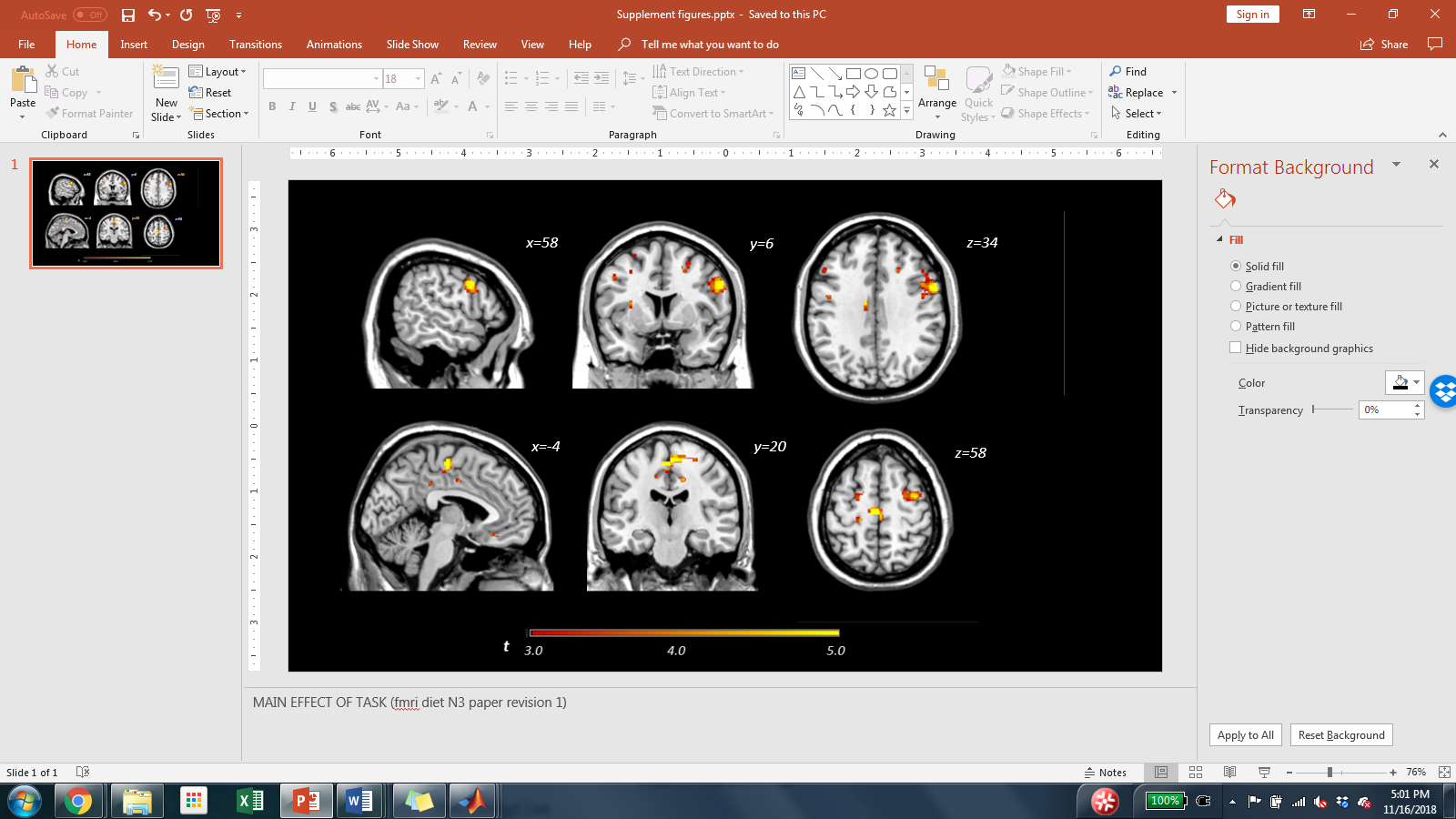


Figure 1. Main effect of task: Activation during successful inhibitions (Correct NoGo>Incorrect NoGo).

Pubertal Development (covariate of no interest) (Table 2, Figure 2)

With the addition of this covariate, the original ACC cluster is reduced in size by 15 voxels (now >95% of original size) at an FWE corrected p=0.005.

| **Region** | ***x*** | ***y*** | ***z*** | **Max *t*** | **Volume (mm3)** |
| --- | --- | --- | --- | --- | --- |
| Inferior frontal gyrus, left | -42 | 0 | 24 | 3.44 | 11 |
| Insula, right | 46 | -2 | -2 | 4.03 | 24 |
| Insula, left | -40 | -2 | 6 | 3.54 | 18 |
|  | -34 | 18 | -4 | 3.52 | 11 |
| Anterior cingulate, left | -14 | 42 | -6 | 3.87 | 14 |
| Cingulate gyrus, right  [BA 32/24]* | 8 | 22 | 30 | 4.29 | 292* |
|  | 2 | 26 | 22 | 3.85 |  |

Table 2. MNI Coordinates of local maxima for activation during successful inhibitions (Correct No-Go>Incorrect No-Go) inversely associated with dietary Omega-3 Index intake, controlling for Pubertal Development Score (cluster defining threshold ke=10, uncorrected p=0.001, df 84). *Group-level statistical maps were small volume corrected (SVC) for multiple comparisons within the PFC. Cluster surviving FWE correction at p<0.05 denoted.


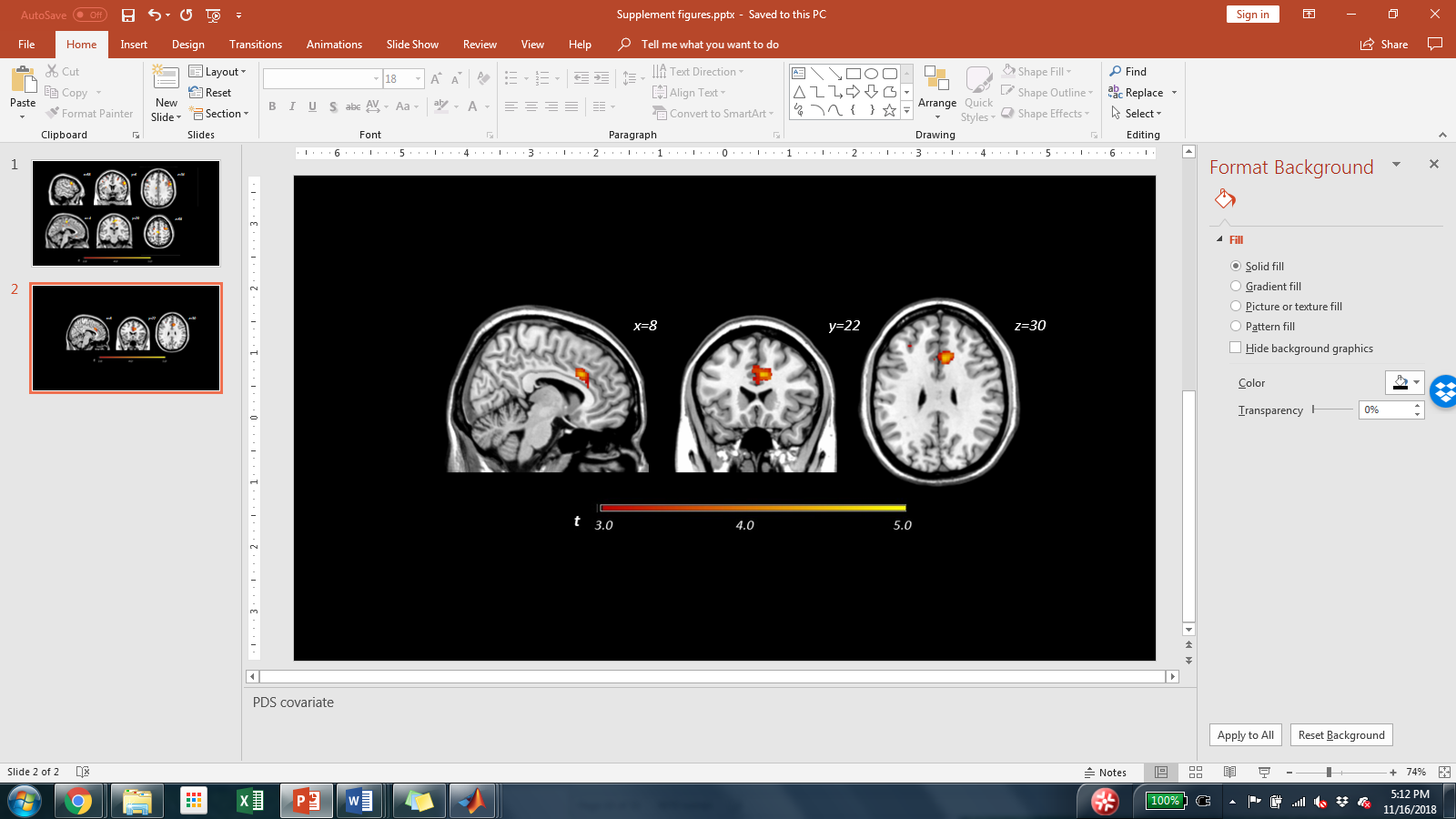


Figure 2. Activation during successful inhibitions (Correct NoGo>Incorrect NoGo) inversely related to dietary Omega-3 index, including Pubertal Development Score as covariate of no interest.
